# Ecology of protection: probiotic biogeography and sepsis prevention in the neonatal intestine

**DOI:** 10.1128/mbio.01127-26

**Published:** 2026-08-10

**Authors:** Sierra C. Hansen, Christopher W. Hamm, Jeffrey R. Singer, Casey T. Weaver, Michael J. Gray

**Affiliations:** 1 Department of Microbiology, Heersink School of Medicine, University of Alabama at Birmingham 9968, Birmingham, Alabama, USA; 2 Department of Pathology, Heersink School of Medicine, University of Alabama at Birmingham 9968, Birmingham, Alabama, USA; Columbia University, New York, New York, USA

**Keywords:** probiotics, neonatal sepsis, microbial ecology

## Abstract

Neonatal infection is a leading cause of morbidity and mortality worldwide, particularly among preterm and low birth weight infants. Probiotic bacteria are widely used in peri- and postnatal care and can reduce neonatal intestinal dysbiosis. However, formulations and efficacy remain highly variable, highlighting a critical gap in our understanding of the mechanisms that drive successful interventions in this population. Furthermore, current studies on probiotic efficacy rely on indirect or relative measures of intestinal bacterial burden. Here, we directly mapped the biogeography of intestinal colonization and quantified the probiotic effects of *Escherichia coli* Nissle 1917 (EcN) and *Ligilactobacillus murinus* strain V10 against *Klebsiella pneumoniae* dysbiosis across the neonatal murine intestine. Despite substantial differences in their spatial distribution along the intestine, both EcN and *L. murinus* V10 significantly reduced *K. pneumoniae* colonization and mortality from *K. pneumoniae* sepsis, with EcN providing greater protection. EcN’s probiotic activity was partially dependent on high-affinity oxygen respiration, implicating luminal oxygen availability as a key ecological determinant of probiotic efficacy. Contrary to the common assumption that multi-strain probiotics are inherently superior, simultaneous administration of EcN and *L. murinus* V10 was less effective than EcN treatment alone at preventing sepsis-related death. These findings identify intestinal niche occupancy, oxygen utilization, and strain-strain interactions as critical variables which should inform the rational design of future probiotic interventions for high-risk neonates.

**IMPORTANCE:**

Late-onset sepsis (LOS) remains a devastating and difficult-to-treat complication of prematurity, and probiotics are increasingly used to reduce dysbiosis and infection risk in this vulnerable population. Probiotic regimens, however, are highly heterogeneous, and their mechanisms of action in the neonatal intestine are poorly defined, complicating efforts to design safe, effective, and regulatable interventions. In this work, we use a neonatal mouse model of LOS to rigorously test fundamental assumptions underlying the current paradigm for understanding the impact of probiotics on intestinal disease. We demonstrate that two distantly related probiotic bacteria, *Escherichia coli* Nissle 1917 and *Ligilactobacillus murinus* V10, each reduce intestinal colonization and mortality caused by the LOS pathobiont *Klebsiella pneumoniae*, but do so through distinct ecological and molecular mechanisms. These findings highlight ecological principles, including spatial niche occupancy, resource competition, and strain-strain interactions, as critical determinants of probiotic efficacy, and provide mechanistic insight that will be important for guiding rational probiotic strategies for high-risk neonates.

## INTRODUCTION

Neonatal infection remains a leading cause of both neonatal morbidity and mortality worldwide, particularly among preterm infants. Preterm infants exhibit unique susceptibility to intestinal dysbiosis (1–3). Dysbiosis is defined here as overgrowth within the neonatal intestine by a single species from one of the families of facultatively anaerobic bacteria associated with neonatal sepsis: Enterococcaceae, Staphylococcaceae, or Enterobacteriaceae (1, 4–6). Risk factors such as premature membrane rupture and intra-amniotic infection are common in this population (1, 3) and can precipitate dysbiosis, while high rates of cesarean delivery (7, 8), prolonged neonatal intensive care unit (NICU) stays (9), and intensive medical interventions further shape early microbiome assembly and infection risk.

The most prominent short-term consequences of dysbiosis at this developmental stage are necrotizing enterocolitis (NEC), a necrotic infection of the large intestine (10), and sepsis, including late-onset sepsis (LOS), defined as sepsis presenting between 72 hours and 28 days after birth (4). Both NEC and LOS remain major drivers of morbidity and mortality in preterm and low birth weight infants (5, 11, 12). Although establishing causality remains challenging, the prevailing clinical hypothesis posits that intestinal dysbiosis enables translocation of gut bacteria into the bloodstream: a notion bolstered by studies matching septic infants’ blood culture isolates to their gut microbiota (13–16), and by work showing that increased colonization by beneficial bacteria can reduce sepsis risk (15, 17).

Probiotic bacteria have been shown to mitigate neonatal intestinal dysbiosis (18–26) and are widely used in peri- and postnatal clinical settings. However, substantial variation in probiotic formulations and reported efficacy reveals critical gaps in the current understanding of the mechanisms governing probiotic efficacy in this population. This gap is further compounded by regulatory frameworks that favor commercial interests over scientific validation. In 2023, the US Food and Drug Administration issued a blanket warning against the use of probiotics in preterm infants, prompted by a single case report of sepsis and death following administration of the probiotic strain *Bifidobacterium longum* subsp. *infantis* EVC001 (27). This warning markedly curtailed probiotic use in US NICUs, despite criticism from multiple groups and organizations (28, 29), including the European Society for Paediatric Gastroenterology, Hepatology and Nutrition (ESPGHAN), and the European Foundation for the Care of Newborn Infants (EFCNI) (29), which advocate for probiotics’ established role in neonatal care. Together, these developments underscore the tension between regulatory caution and existing clinical data and highlight the need for mechanistic studies to guide safe and effective probiotic strategies.

Most prior work on neonatal probiotics has relied on indirect or relative measures of bacterial burden, such as fecal microbiome profiling or metabolite quantification, which provide limited spatial resolution and can obscure the ecological processes that govern probiotic-pathobiont interactions. In addition, fundamental questions remain about how specific probiotic strains colonize the neonatal intestine, how host-associated factors such as luminal oxygen availability shape their activity, and whether combining multiple probiotic species yields additive, synergistic, or even antagonistic effects.

In this study, we directly quantified the probiotic effects of two distinct bacterial species, *Escherichia coli* Nissle 1917 (EcN 1917) and *Ligilactobacillus murinus* V10 (V10), against the pathobiont *Klebsiella pneumoniae* in a neonatal mouse model. By mapping spatial colonization patterns across intestinal segments, exploiting EcN respiratory mutants as *in vivo* biosensors of luminal oxygen, and testing both single- and dual-species probiotic regimens, we sought to define how ecological factors such as niche occupancy, oxygen utilization, and priority effects shape probiotic efficacy against dysbiosis and sepsis. The results of these experiments demonstrated that targeted probiotic interventions effectively reduce dysbiosis and sepsis mortality through compartment-specific ecological and molecular mechanisms, and that, contrary to the dominant paradigm in the field, combining multiple probiotics does not necessarily lead to increased protective effects.

## RESULTS

Much of the intestinal development that takes place *in utero* in humans occurs postnatally in rodents (30), meaning that the newborn mouse is more similar developmentally to a preterm than a full-term human neonate. Like preterm human infants, neonatal mice also exhibit delayed colonization by obligate anaerobes (17). To model dysbiosis and late-onset sepsis, we employed a mouse model previously established by our laboratory (17), in which we orally gavage neonatal (5–7 days old, depending on the experiment) mice with strains of *Klebsiella pneumoniae*, a clinically significant pathogen in late-onset sepsis (6). Engineered bioluminescent *K. pneumoniae* strains enable *in vivo* visualization and approximate quantification of infection dynamics in live mice in this model (17). However, most experiments in this paper instead relied on direct plate counts for precise enumeration of viable bacterial burdens in whole or segmented neonatal intestines. We utilized two *K. pneumoniae* strains: *Kp-39* and *Kp-43816*^lux^ (17). Notably, *Kp-39* does not induce sepsis in neonatal mice (17), allowing these experiments to specifically evaluate probiotic efficacy in curbing dysbiotic intestinal overgrowth of *K. pneumoniae*. In contrast, the more virulent *Kp-43816*^lux^ strain provokes fulminant sepsis and death in our model (17).

### EcN 1917 reduces *Kp-39* colonization burden across the intestinal span

**Fig 1:**
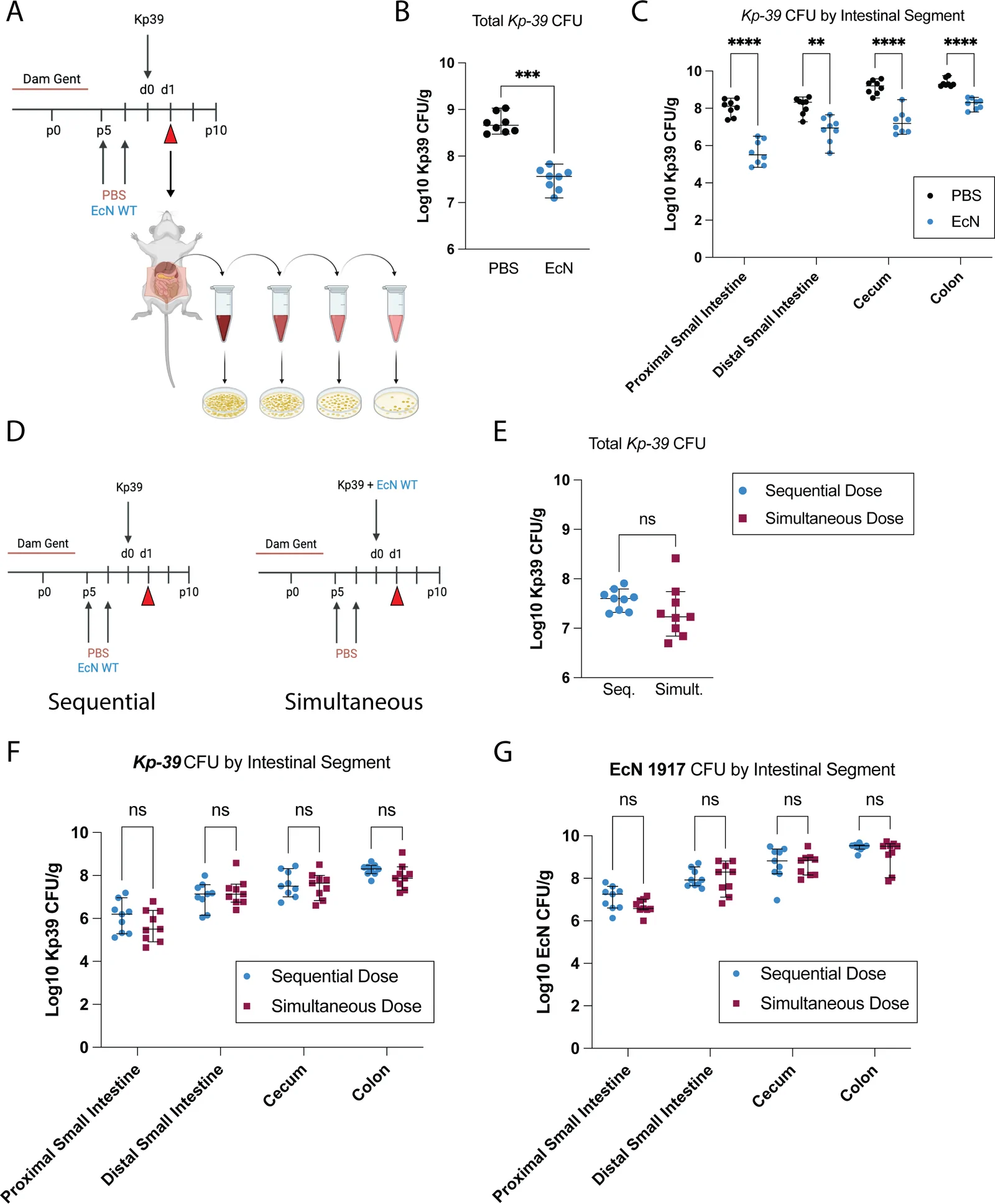
Biogeography of EcN 1917 probiotic effect in the neonatal intestine. (**A**) Schematic illustration of murine model of neonatal sepsis with probiotic treatment. Littermate pups from gentamicin-treated dams were treated intragastrically (i.g.) on days 5 (P5) and 6 (P6) of life with either EcN 1917 or PBS control before infection with 10^7^ CFU *Kp-39* on day 7 (p7/d0), then sacrificed on d1 (indicated with a red arrow; extra graphics from BioRender). (**B**) Total intestinal *Kp-39* colonization burden by treatment group on treatment day 1 (D1). (**C**) *Kp-39* colonization by treatment group and intestinal segment on d1. Data were pooled from two independent experiments of within-litter controls with PBS (*n* = 8) and EcN (*n* = 8). (**D**) Schematic illustration of neonatal priority effect model. Littermate pups from gentamicin-treated dams were treated i.g. on days 5 (P5) and 6 (P6) of life with either EcN 1917 (sequential dose) or PBS control (simultaneous dose) before infection with 10^7^ CFU *Kp-39* (sequential dose) or 10^7^ CFU *Kp-39* and EcN 1917 (simultaneous dose) on day 7 (p7/d0) and sacrificed on d1. (E) Total intestinal *Kp-39* colonization burden by treatment group on treatment day 1 (D1). (F) *Kp-39* colonization by treatment group and intestinal segment on d1. Data were pooled from two independent experiments of within-litter controls with sequential dose (*n* = 9) and simultaneous dose (*n* = 9). In all instances, *n* refers to the number of pups of either sex. Median values are plotted with error bars indicating 95% CI. Mann-Whitney test (**B and E**) or two-way ANOVA with Sídák’s multiple comparisons test (**C, F, and G**): **P ≤ 0.005; ***P ≤ 0.0005; ****P ≤ 0.0001; ns, not statistically significant. All CFU/g values are determined from plating the entire homogenate of each intestinal segment.

Adult mice are resistant to colonization by probiotics (31), but this is not true of neonatal mice (17). Probiotic administration of EcN 1917 (Fig. 1A) to neonatal mice significantly reduced total intestinal *Kp-39* colonization burden, measured as colony-forming units (CFU) per gram across all segments combined (Fig. 1B), through marked reductions in each of the four intestinal segments examined (Fig. 1C).

### EcN 1917 probiotic efficacy is independent of priority effect

Priority effects, whereby the influence a species has on its environment depends on arrival order, are well documented across microbial and macroscopic ecosystems (32–35). In our model, however, EcN 1917-mediated suppression of *Kp-39* colonization did not depend on dosing sequence (Fig. 1D through G). Littermates receiving EcN 1917 prior to or simultaneously with *Kp-39* showed comparable total (Fig. 1E) and segmental (Fig. 1F) *Kp-39* colonization burdens to those dosed simultaneously. Both dosing regimens equally supported EcN 1917 colonization across intestinal segments (Fig. 1G). These data indicate that the probiotic activity of EcN 1917 is insensitive to priority effects in this setting.

### Luminal oxygen content decreases over the first 2 weeks of normal development

Susceptibility to neonatal dysbiosis and sepsis declines with postnatal age and increasing obligate anaerobe abundance, and in our *Kp-43816*^lux^ sepsis model, resistance emerges by postnatal day 14 (p14) (17).

Direct measurement of intestinal oxygen concentration in neonatal mouse intestines remains technically challenging (36). We therefore adapted a competitive index (CI) assay (37–43) using EcN 1917 respiratory mutants with defined defects in high- or low-affinity oxygen respiration (44–46) or anaerobic respiration (47–52) as *in vivo* biosensors.

**Fig 2:**
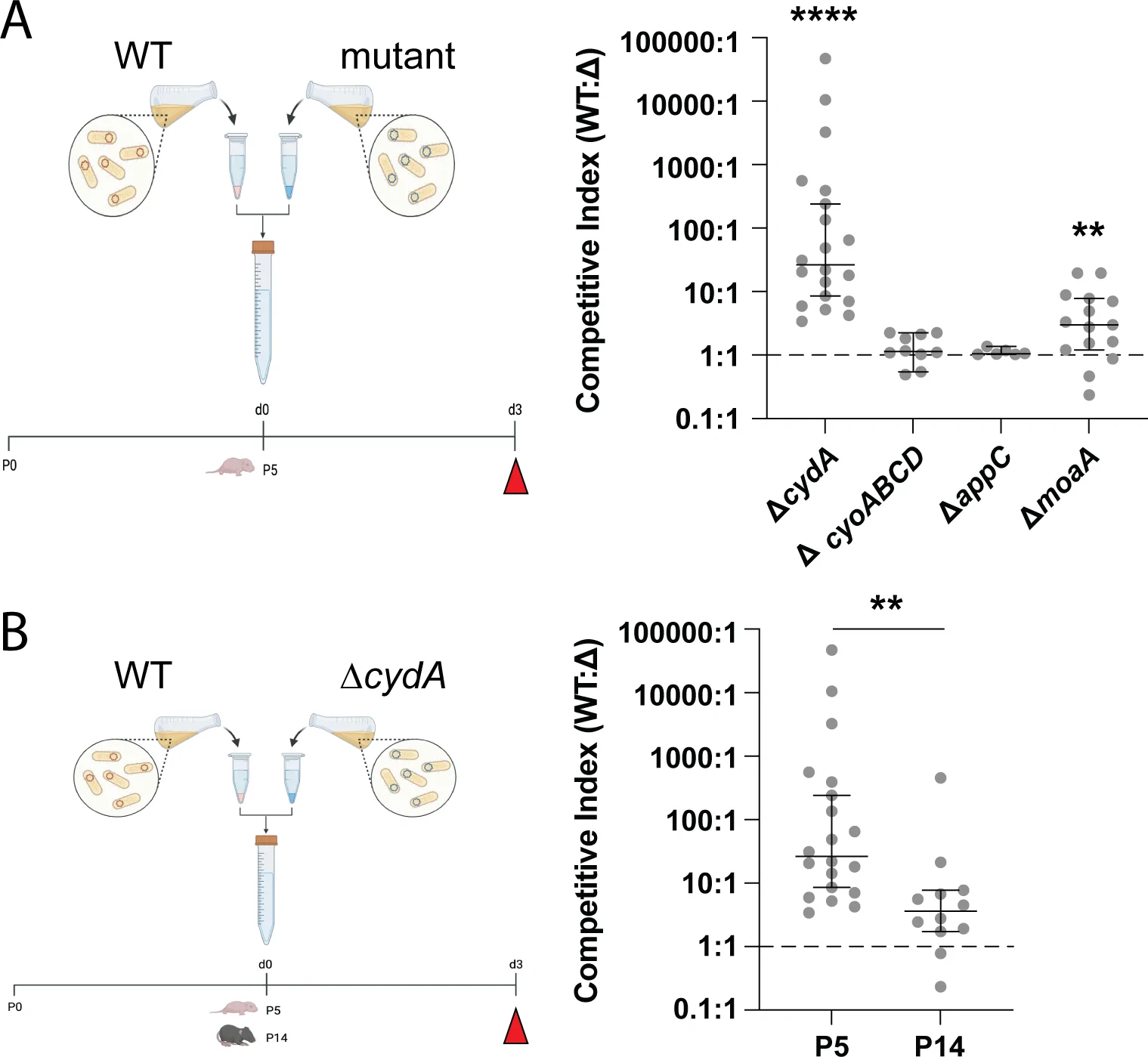
The p5 intestinal lumen is a microaerophilic environment and colonic luminal oxygen levels decrease over the first 2 weeks of normal development. (**A**) p5 SPF mice (*n* = 6–20 per mutant) were inoculated with a 1:1 mixture of *E. coli* WT and *cydA*, *cyoABCD*, *appC,* or *moaA* mutants. The CI (CFU of wild type/CFU of mutant) was determined 3 days after inoculation. (**B**) p5 and p14 SPF mice (*n* = 16–20) were inoculated with a 1:1 mixture of EcN 1917 WT and cydA mutant (∆cydA). The CI was determined 3 days after inoculation. Median values are plotted with error bars indicating 95% CI. Kruskal-Wallis test with Dunn’s multiple comparisons test (**A**): ****P ≤ 0.0001, **P ≤ 0.01; Mann-Whitney test (**B**): **P ≤ 0.01.

At postnatal day 5 (p5), wild-type (WT) EcN 1917 outcompeted the high-affinity ∆*cydA* mutant and, to a lesser degree, the anaerobic ∆*moaA* respiratory mutant in the colon, whereas competition with low-affinity oxidase mutants ∆*appC* or ∆*cyoABCD* showed no difference, consistent with microaerophilic luminal conditions (Fig. 2A).

This competitive advantage was abrogated by postnatal day 14 (p14) (Fig. 2B), indicating a shift to anaerobic conditions, which is perhaps expected given the concurrent increase in colonization by obligate anaerobes previously described at this timepoint (17). This transition in luminal oxygen temporally aligns with dysbiosis resistance, suggesting reduced luminal oxygen may protect against the development of dysbiosis.

### Deletion of the high-affinity oxidase CydA reduces EcN 1917 probiotic effect against *Kp-39* dysbiosis in the distal small intestine and colon

**Fig 3:**
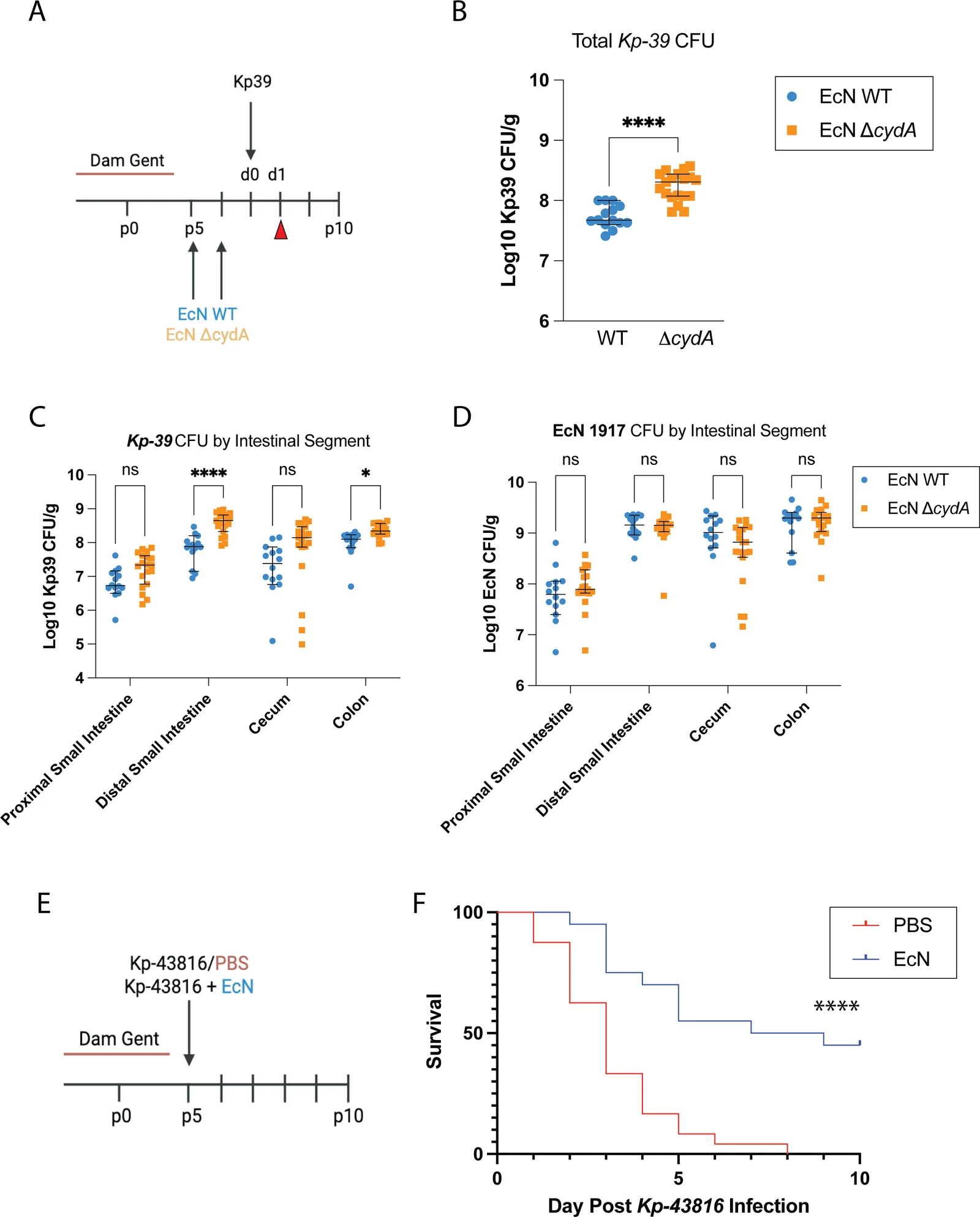
Probiotic effect of EcN 1917 is partially dependent on its ability to respire oxygen using its high-affinity cytochrome oxidase CydA and extends survival against *Kp* sepsis when administered as a probiotic on P5. (**A**) Schematic illustration of neonatal probiotic model. Littermate pups from gentamicin-treated dams were treated i.g. on days 5 (P5) and 6 (P6) of life with either EcN 1917 WT or EcN 1917 ∆cydA before infection with 10^7^ CFU *Kp-39* on day 7 (P7). (**B**) Total intestinal *Kp-39* colonization burden by treatment group on treatment day 1 (D1). (**C**) *Kp-39* colonization by treatment group and intestinal segment on d1. (**D**) EcN 1917 colonization by treatment group and intestinal segment on d1. Data were pooled from three independent experiments of within-litter controls with WT (*n* = 14) and ∆c*ydA* (*n* = 19). In all instances, *n* refers to the number of pups of either sex. Median values are plotted with error bars indicating 95% CI. Mann-Whitney test (**B**) or two-way ANOVA (**C and D**): *P ≤ 0.05; ****P ≤ 0.0001; ns, not statistically significant. (**E**) Dams were separated 1–2 days before delivery and drinking water was supplemented with gentamicin (gent) until p4. On p5, pups were administered 10^7^ CFU *Kp-43816* with 10^7^ CFU EcN 1917 or without probiotic as a PBS negative control and monitored daily for sepsis. (**F**) Kaplan-Meier curve. For survival analysis, data were pooled from multiple independent experiments with PBS (*n* = 24) and EcN (*n* = 20). In all instances, *n* refers to the number of pups of either sex. Log-rank (Mantel-Cox) test: ****P ≤ 0.0001. All CFU/g values are determined from plating the entire homogenate of each intestinal segment.

In addition to its established role as a biosensor of intestinal oxygen content, EcN 1917 functions as a potent probiotic in this model system (Fig. 1). However, the extent to which EcN 1917’s respiratory activity contributes to its inhibition of *Kp-39* colonization remained unclear. In adult mice, oxygen respiration by EcN 1917 is known to be required for inhibition of pathogenic facultative anaerobes, likely through competition for respiratory substrates (37, 42, 43). To assess the contribution of high-affinity oxygen respiration mediated by the CydA oxidase to EcN 1917’s probiotic activity against *Kp-39* colonization in the neonatal intestine, littermate pups were administered either wild-type or ∆*cydA* EcN 1917 prior to challenge with *Kp-39* (Fig. 3A).

Treatment with EcN 1917 ∆*cydA* resulted in higher total intestinal *Kp-39* colonization burden compared to treatment with wild-type EcN 1917 (Fig. 3B), indicating a role for oxygen respiration in probiotic function. However, this difference was not broadly distributed throughout the intestine but was specifically attributable to increased *Kp-39* colonization in the distal small intestine and colon (Fig. 3C). Importantly, there was no difference in the ability of wild-type and Δ*cydA* EcN 1917 to colonize any intestinal segment (Fig. 3D), so the reduced probiotic efficacy of the ∆*cydA* mutant is not due to it simply being less abundant. The defect must therefore be due to the lack of CydA activity, with the simplest hypothesis being that wild-type EcN 1917 outcompetes *Kp-39* for O_2_ via CydA-dependent respiration. The Δ*cydA* mutant still reduced total intestinal *Kp-39* colonization relative to PBS controls (Fig. 3B), so the ability of EcN 1917 to reduce *Kp-39* colonization cannot be fully explained by direct competition for oxygen between these facultative anaerobes.

### EcN 1917 supplementation extends survival in *K. pneumoniae* sepsis

Given the well-established role of the microbiome in mitigating neonatal sepsis risk through prevention of dysbiosis (17), we next sought to determine whether the protective effects observed in the *Kp-39* dysbiosis model extended to the prevention of fulminant sepsis caused by the more virulent *K. pneumoniae* strain *Kp-43816*^lux^.

Previous studies evaluating probiotic prophylaxis against *Kp-43816*^lux^-induced sepsis were confounded by high litter mortality following the standard three-dose gavage protocol (17), which was likely attributable to bloodstream invasion resulting from minor gavage-associated microtrauma. Given that natural bloodstream invasion by intestinal pathogens requires epithelial translocation (53), this experimental mortality likely overestimates the clinical severity of infection. Having established equivalent reduction of *Kp-39* colonization with simultaneous and sequential EcN 1917 / *Kp-39* dosing (Fig. 1E and F), the sepsis model was therefore refined to reduce procedural trauma by consolidating dosing into a single gavage.

Using this revised approach, litters of gentamicin-reared pups were administered *Kp-43816*^lux^ simultaneously with either EcN 1917 or a PBS vehicle control and monitored daily for mortality (Fig. 3E). Probiotic supplementation with EcN 1917 produced a striking survival benefit compared to PBS treatment (Fig. 6B), with 50% of EcN 1917-treated pups surviving to day 10 post-infection, whereas all PBS-treated controls succumbed to infection (Fig. 3F, *P* < 0.0001). These results demonstrate that EcN 1917 supplementation confers significant protection against lethal *K. pneumoniae* sepsis, likely via early stabilization of intestinal microbial communities that limit pathogen invasion.

### *L. murinus* V10 reduces *Kp-39* colonization burden across the intestinal span

**Fig 4:**
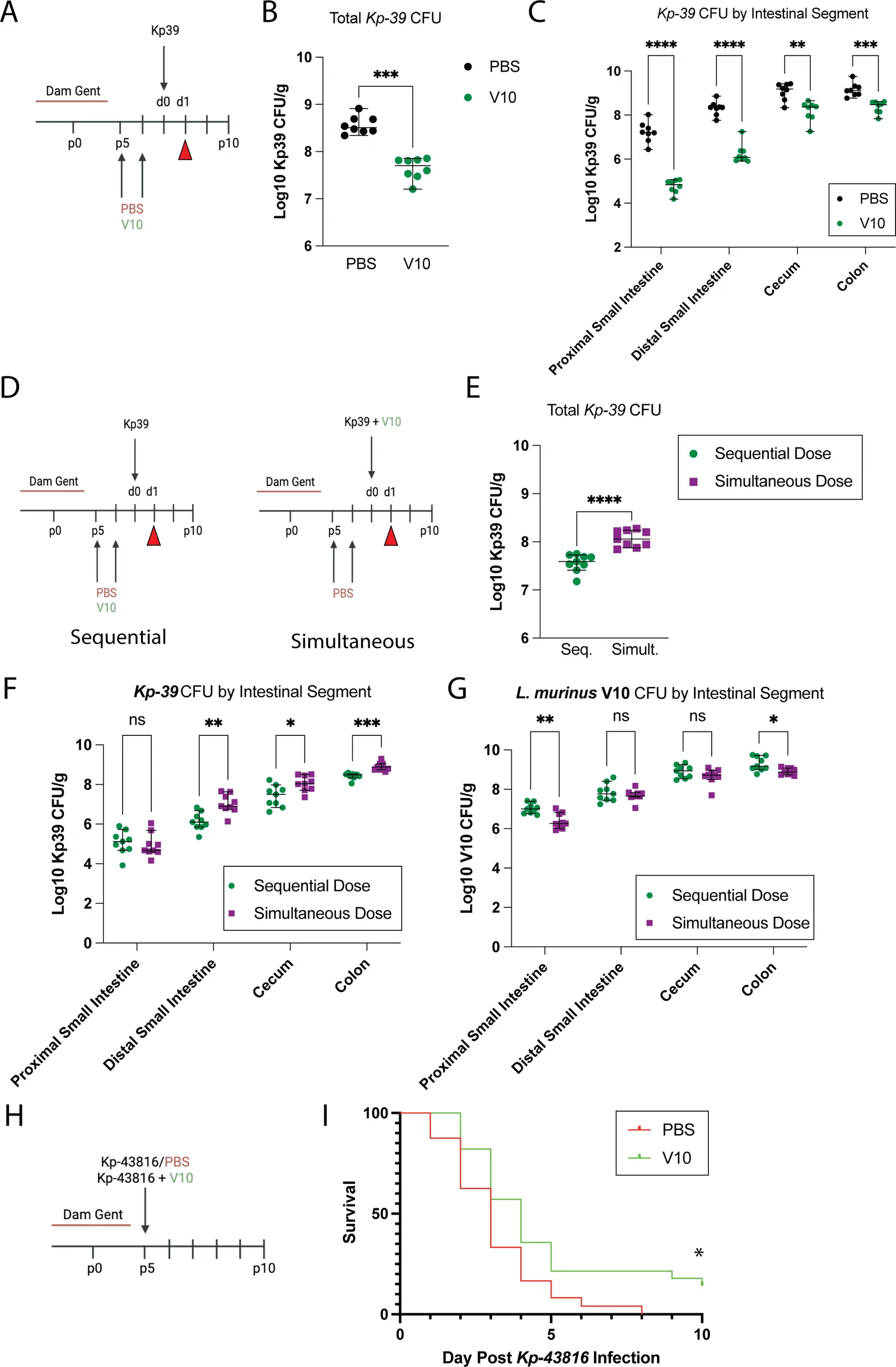
Biogeography and priority effect of *L. murinus* V10 probiotic effect in the neonatal intestine. (**A**) Schematic illustration of neonatal probiotic model. Littermate pups from gentamicin-treated dams were treated i.g. on days 5 (P5) and 6 (P6) of life with either *L. murinus* V10 or PBS control before infection with 10^7^ CFU *Kp-39* on day 7 (P7/d0) and sacrificed on d1. (**B**) Total intestinal *Kp-39* colonization burden by treatment group on treatment day 1 (D1). (**C**) *Kp-39* colonization by treatment group and intestinal segment on d1. Data were pooled from two independent experiments of within-litter controls with PBS (*n* = 8) and V10 (*n* = 8). (**D**) Schematic illustration of neonatal priority effect model. Littermate pups from gentamicin-treated dams were treated i.g. on days 5 (P5) and 6 (P6) of life with either *L. murinus* V10 (sequential dose) or PBS control (simultaneous dose) before infection with 10^7^ CFU *Kp-39* (sequential dose) or 10^7^ CFU *Kp-39* and *L. murinus* V10 (simultaneous dose) on day 7 (P7). (E) Total intestinal *Kp-39* colonization burden by treatment group on treatment day 1 (D1). (F) *Kp-39* colonization by treatment group and intestinal segment on d1. (H) *L. murinus* V10 colonization by treatment group and intestinal segment on d1. Data were pooled from two independent experiments of within-litter controls with sequential dose (*n* = 9) and simultaneous dose (*n* = 9). (G) Dams were separated 1–2 days before delivery and drinking water was supplemented with gentamicin (gent) until p4. On p5, pups were administered 10^7^ CFU *Kp-43816* with 10^7^ CFU *L. murinus* V10 or without probiotic as a PBS negative control and monitored daily for sepsis. (H) Kaplan-Meier curve. For survival analysis, data were pooled from multiple independent experiments with PBS (*n* = 24) and V10 (*n* = 28). In all instances, *n* refers to the number of pups of either sex. Median values are plotted with error bars indicating 95% CI. Mann-Whitney test (**B and E**), two-way ANOVA (**C, F, and G**), or log-rank (Mantel-Cox) test (**H**): *P ≤ 0.05; **P ≤ 0.005; ***P ≤ 0.0005; ****P ≤ 0.0001; ns, not statistically significant. All CFU/g values are determined from plating the entire homogenate of each intestinal segment.

*Ligilactobacillus murinus* is a well-characterized intestinal commensal in mice and other rodents, but it is not typically found as a member of the human microbiome (17, 44, 45). In prior work (17), we isolated a strain of *L. murinus,* designated V10, from the intestinal microbiome of neonatal mice that were protected against *K. pneumoniae* dysbiosis. When administered as a probiotic, *L. murinus* V10 protects against the development of *Kp-39* dysbiosis in the neonatal murine intestine and is associated with reduced incidence of late-onset sepsis in the same population (17). Consistent with these previous observations, probiotic supplementation with *L. murinus* V10 (Fig. 4A) decreased total intestinal *Kp-39* colonization (Fig. 4B), reflecting a global reduction in colonization burden across all four examined intestinal segments relative to PBS vehicle controls (Fig. 4C). In line with our findings for *L. murinus* V10, diverse *Lactobacillus* spp. are known to colonize the full length of the intestine (46, 47). However, unlike the obligate anaerobes that typically dominate the colon, lactobacilli are efficient colonizers of the small intestine (48), so it is perhaps unsurprising that the largest and most significant changes in *Kp-39* colonization following probiotic intervention with *L. murinus* V10 are observed in the proximal intestinal segments.

### Prior exposure to *L. murinus* V10 enhances its probiotic effect

To evaluate the impact of priority effect on the ability of *L. murinus* V10 to reduce *Kp-39* colonization burden, littermate pups were administered the probiotic either according to the standard sequential dosing schedule or simultaneously with the *Kp-39* infectious challenge (Fig. 4D). In contrast to our observations with EcN 1917 (Fig. 1), *L. murinus* V10 exhibited a clear timing dependence: sequential dosing reduced total intestinal *Kp-39* burden more than simultaneous dosing with the largest differences in the distal small intestine, cecum, and colon (Fig. 4E and F). Although dosing schedule also affected *L. murinus* V10 colonization, the segments with reduced V10 abundance (the proximal small intestine and colon) did not precisely match those with increased *Kp-39* microbial load (the distal small intestine, cecum, and colon) (Fig. 4G), suggesting that local probiotic abundance alone does not fully explain its protective effect.

### Simultaneous *L. murinus* V10 supplementation increases survival during *K. pneumoniae* sepsis challenge

The priority effect observed for *L. murinus* V10 (Fig. 4D through G) complicated direct assessment of how well it protects against *Kp-43816*^lux^ sepsis. As noted above, the multiple gavages required for sequential dosing of probiotic and *K. pneumoniae* led to substantial mortality, likely due to bloodstream infection arising from gavage-associated microtraumas in the oropharynx. Consequently, we were unable to directly test whether the dosing regimen that most effectively limited *Kp-39* dysbiosis would also be superior in preventing *Kp-43816*^lux^ sepsis. However, given that simultaneous administration of *L. murinus* V10 and *Kp-39* colonization still reduced *Kp-39* colonization compared to PBS alone (Fig. 4E vs Fig. 4B), we adopted a single-gavage strategy for the virulent *Kp-43816*^lux^ in combination with *L. murinus* V10.

As above with EcN 1917, in this adapted model, litters of gentamicin-reared pups received *Kp-43816*^lux^ simultaneously with either *L. murinus* V10 or a PBS vehicle control and were monitored daily for mortality (Fig. 4H). Probiotic supplementation with *L. murinus* V10 produced a modest but statistically significant survival benefit compared to PBS vehicle alone (*P* = 0.011) (Fig. 4I). Notably, the magnitude of this survival advantage was substantially less than that observed with EcN 1917 under analogous conditions (Fig. 3F), suggesting that while *L. murinus* V10 provides measurable protection against fulminant *K. pneumoniae* sepsis, its protective capacity is more limited than that of EcN 1917.

### Potential *L. murinus* V10 probiotic action

While *L. murinus* V10 was able to colonize all segments of the neonatal mouse intestine, it exerted its strongest inhibitory effects on *Kp-39* in the proximal segments (Fig. 4C), where oxygen availability is highest (49). Using EcN 1917 as a biosensor, we demonstrated that the neonatal intestine contains substantial oxygen at early time points (Fig. 2), when animals are most susceptible to dysbiosis. Together with our prior observation that protection from dysbiosis correlates with both colonization by anaerobic bacteria and increased signal from a hypoxia-sensing probe in the neonatal mouse intestine (17); these findings led us to hypothesize that oxygen-dependent responses may also help explain the probiotic activity of *L. murinus* V10.

**Fig 5:**
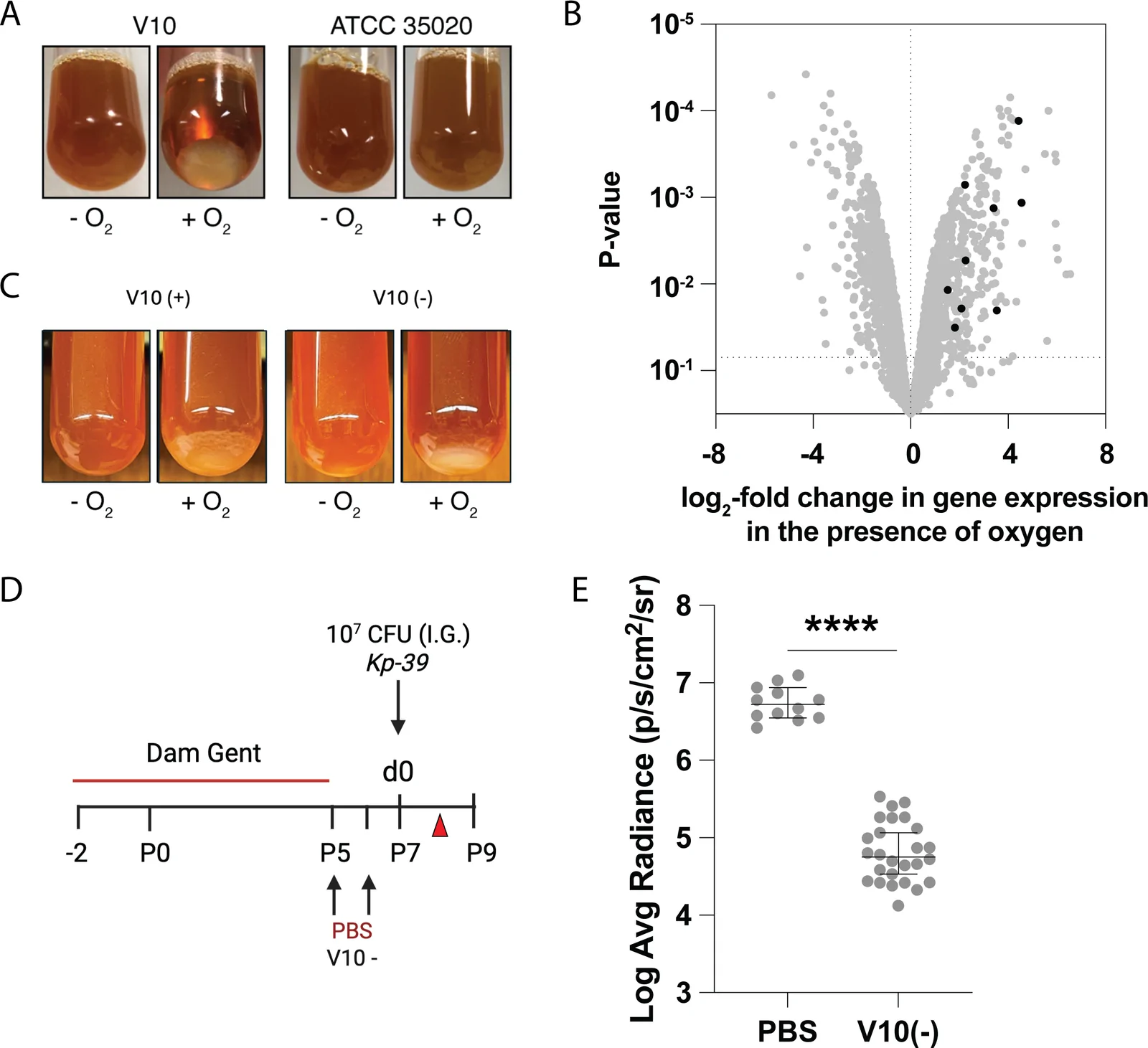
Megaplasmid pV10 is not responsible for the probiotic phenotype of *L. murinus* strain V10. (**A**) *L. murinus* strains V10 and ATCC 35020 were incubated for 5 h at 37°C in MRS medium either aerobically or anaerobically and then mixed by manual agitation. (**B**) *L. murinus* V10 was grown to *A*_600_ = 2.0 in MRS medium either aerobically or anaerobically, then RNA was extracted and sequenced (*n* = 3 biological replicates per condition) (SeqCenter, LLC). For each gene, log_2_ fold change in average gene expression in aerobic cultures *versus* anaerobic cultures was plotted against the *P*-value for differential gene expression. Black dots indicate predicted highly expressed surface proteins (see Table S3). (**C**) *L. murinus* strains V10 (+) and *L. murinus* V10 (−) were incubated for 5 h at 37°C in MRS medium either aerobically or anaerobically, then mixed by manual agitation. (**D**) Schematic illustration of neonatal probiotic model. Littermate pups from gentamicin-treated dams were treated i.g. on days 5 (P5) and 6 (P6) of life with either *L. murinus* V10 (−) or PBS control before infection with 10^7^ CFU *Kp-39* on day 7 (P7). (E) *Kp-39* average radiance by treatment group (p/s/cm^2^/sr) 1 day post-challenge (red arrow in 5D). Data were pooled from two independent experiments of within-litter controls with PBS (*n* = 10) and V10 (−) (*n* = 18). In all instances, *n* refers to the number of pups of either sex. Median values are plotted with error bars indicating 95% CI. Mann-Whitney test (**E**): ****P ≤ 0.0001.

With this hypothesis in mind, we observed a striking phenotypic difference between *L. murinus* strain V10 and the non-protective *L. murinus* type strain ATCC 35020 (17). When grown in the presence of oxygen, *L. murinus* V10 aggregates and forms a dense, glutinous cell pellet (Fig. 5A), a phenotype that is not observed in anaerobically grown V10 cultures or in cultures of *L. murinus* ATCC 35020. Transcriptomic analysis of *L. murinus* V10 grown with and without oxygen revealed extensive changes in gene expression across the genome, with 815 of 2,484 annotated protein-coding genes significantly different between the two conditions (Data Set S1). These included upregulation of several YSIRK-type signal peptide-containing cell surface proteins that could be involved in aggregation or adhesion (50–52) (Fig. 5B; Tables S1 to S3). Neither transcriptional profiling nor comparative genomics, however, can definitively identify which specific gene(s) in *L. murinus* V10 underlie either the aggregation phenotype or its probiotic activity. Unlike the readily manipulated EcN 1917, molecular genetic tools for *L. murinus* have not yet been reported, making direct experimental validation of these candidate determinants challenging.

Comparative genomics revealed that *L. murinus* V10 harbors a unique 110-kbp megaplasmid, eponymously termed “pV10” (GenBank accession no. CP040853.1), which encodes multiple predicted surface and S-layer proteins (54, 55), making it an attractive candidate for mediating V10’s oxygen-dependent aggregation phenotype and, possibly, probiotic function. To test the contribution of pV10 to the *L. murinus* aggregation phenotype, we used novobiocin to cure *L. murinus* V10 of this plasmid (56, 57), generating a plasmid-free derivative referred to as *L. murinus* V10 (−). *L. murinus* V10 (−) demonstrated a similar degree of oxygen-dependent auto-aggregation to that of the parental V10 strain harboring pV10 (V10 [+]), indicating that the pV10 plasmid is not required for this *in vitro* phenotype (Fig. 5C). Because auto-aggregation and probiotic effectiveness are not necessarily linked, the plasmid-cured *L. murinus* V10 (−) strain presented a unique opportunity to assess the impact of simultaneously eliminating 110 genes on *L. murinus* V10’s ability to protect against *Kp-39* dysbiosis *in vivo*. To this end, littermate pups were administered either *L. murinus* V10 (−) or PBS vehicle control prior to *Kp-39* challenge. To determine whether the plasmid-free strain had any ability to reduce *Klebsiella* burden in live mice, we leveraged the bioluminescence of *Kp-39* as a rapid assessment tool, as described in our previous work (17) (Fig. 5D). *L. murinus* V10 (−) significantly reduced *Kp-*39 burden relative to PBS vehicle controls (Fig. 5E), similar to *L. murinus* V10 (+) (17), indicating that pV10 is not required for the probiotic effect of *L. murinus* V10 against dysbiotic *Kp-39* expansion in the neonatal murine intestine. Given the clear lack of dependence on pV10 for probiotic effect, we did not sacrifice additional animals for more labor-intensive quantitation of viable bacterial counts for this comparison.

### Microbiome analysis of probiotic-treated mice

**Fig 6:**
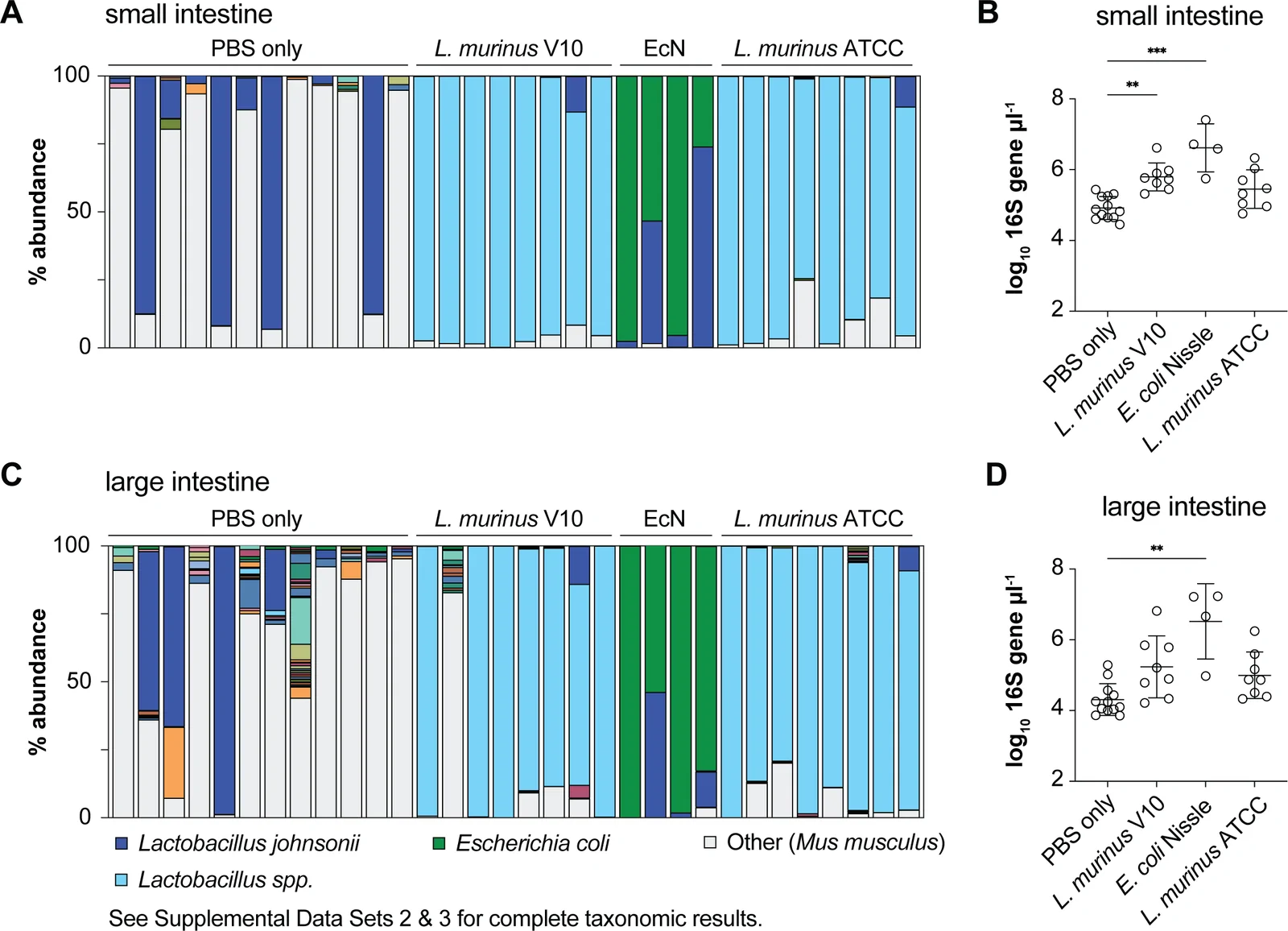
Microbiome analysis of probiotic-treated neonatal mice. 16S rRNA amplicon sequencing of small (**A and B**) and large (**C and D**) intestines from neonatal mice gavaged with PBS, *L. murinus* V10, *E. coli* Nissle 1917 (EcN), or *L. murinus* ATCC 35020 (*L. murinus* ATCC) on p5 and p6, harvested on d0 (P7). Panels A and C show relative abundance of individual species (dark blue = *L. johnsonii*, light blue = unassigned *Lactobacillus* spp. [e.g., *L. murinus*], dark green = *E. coli*, light gray = *Mus musculus* mouse DNA; see Data Sets S2 and S3 for abundance of other species) in the small and large intestines, respectively. Panels B and D show the absolute abundance of reads in each set of samples, determined by qPCR, reported as the log_10_ of the number of reads per µL of extracted DNA (Zymo Research). Kruskal-Wallace test (one-way ANOVA with ranks) with Dunn’s multiple comparisons test: **P ≤ 0.01, ***P ≤ 0.001.

To characterize the impact of probiotic supplementation on the neonatal intestinal microbiota, we used 16S rRNA amplicon sequencing (Zymo Research) (58) on small and large intestinal contents from pups gavaged with PBS, *L. murinus* V10, EcN 1917, or *L. murinus* ATCC 35020 (see timeline in Fig. 1A). The main findings of this analysis are summarized in Fig. 6, with complete data provided in Data Sets S2 and S3. As in prior work (17), the microbiome of PBS-treated mice was sparse both in the small (Fig. 6A) and large (Fig. 6C) intestines: in most animals, the majority of reads mapped to the mouse genome rather than to bacteria (reported as “Other” by the analysis pipeline; see Materials and Methods), and total bacterial read abundance was significantly lower than in probiotic-treated samples (Fig. 6B and D). The most abundant bacterial species detected in PBS-treated mice was *Lactobacillus johnsonii* (59). Seven of 12 PBS-treated mice had detectable *E. coli* in their large intestinal microbiomes, at relative abundances of 0.4%–1.9% of total reads (Data Set S3). No other Enterobacteriaceae were detected in PBS-treated mice. As described in Materials and Methods, fresh breeder mice were obtained from the Jackson Laboratory for each experiment, so this reflects the natural microbiota of those animals, which are reported to contain very low levels of both Enterobacteriaceae and *L. murinus* (60, 61).

**Fig 7:**
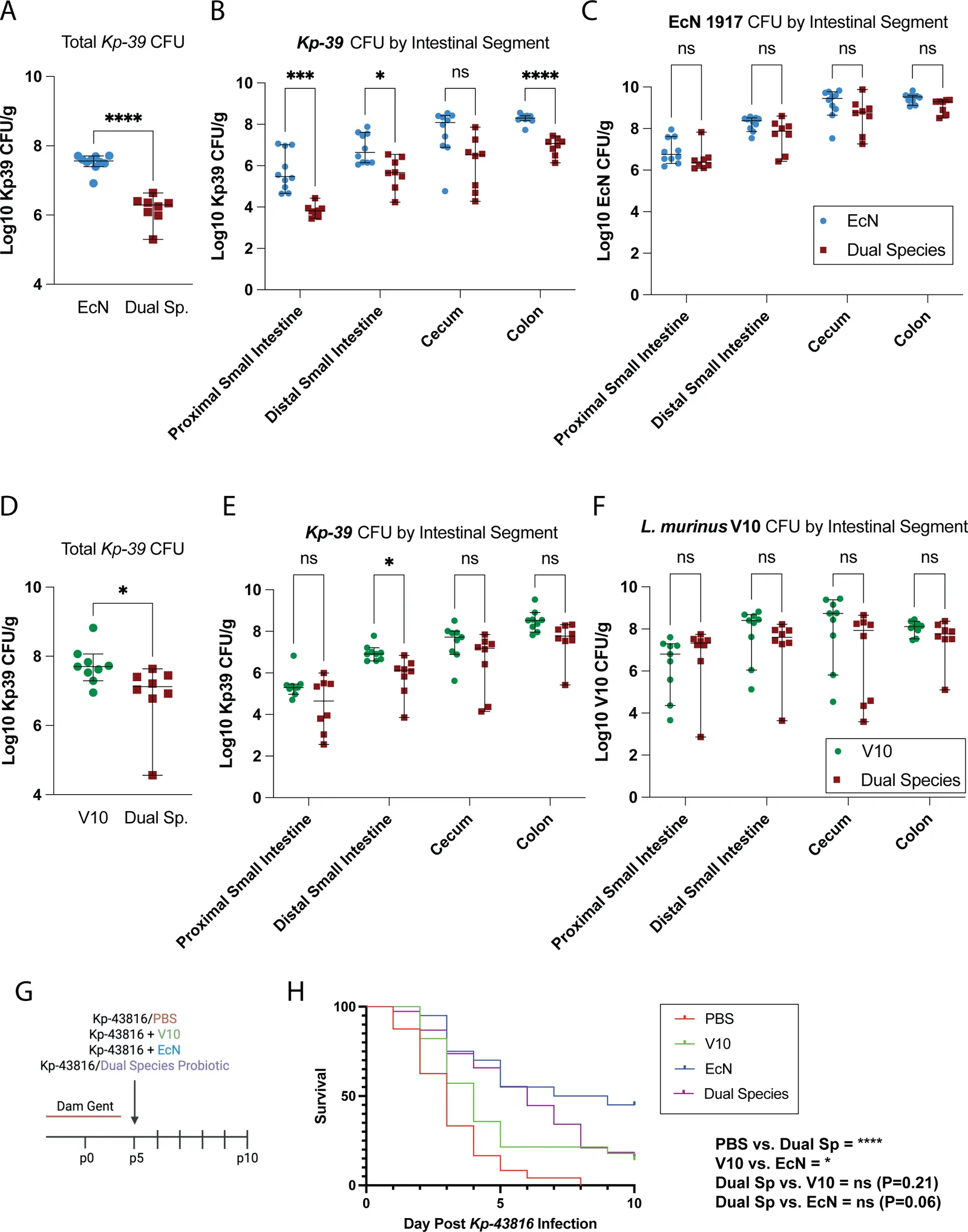
Biogeography of a *L. murinus* V10/EcN 1917 dual-species probiotic effect in the neonatal intestine. (**A**) Total intestinal *Kp-39* colonization burden by treatment group on treatment day 1 (D1). (**B**) *Kp-39* colonization by treatment group and intestinal segment on d1 and intestinal segment on d1. (**C**) EcN 1917 colonization by treatment group and intestinal segment on d1. (**D**) Total intestinal *Kp-39* colonization burden by treatment group on treatment day 1 (D1). (**E**) *Kp-39* colonization by treatment group and intestinal segment on d1. (**F**) *L. murinus* V10 colonization by treatment group and intestinal segment on d1. Data were pooled from two independent experiments of within-litter controls with EcN (*n* = 10) and its dual-species control (*n* = 8), V10 (*n* = 9) and its dual-species control (*n* = 8). (**G**) Dams were separated 1–2 days before delivery, and drinking water was supplemented with gentamicin (gent) until p4. On p5, pups were administered 10^7^ CFU *Kp-43816* with 10^7^ CFU EcN 1917 or *L. murinus* V10 or without probiotic as a PBS negative control and monitored daily for sepsis. (**H**) Kaplan-Meier curve. For survival analysis, data were pooled from multiple independent experiments with PBS (*n* = 24), EcN (*n* = 20), V10 (*n* = 28), and dual species (*n* = 38). In all instances, *n* refers to the number of pups of either sex. Median values are plotted with error bars indicating 95% CI. Mann-Whitney test (**A and D**) or two-way ANOVA (**B, C, E, and F**) or log-rank (Mantel-Cox) test (**H**): *P ≤ 0.05; ***P ≤ 0.0005; ****P ≤ 0.0001; ns, not statistically significant. All CFU/g values are determined from plating the entire homogenate of each intestinal segment.

Following probiotic gavage, overall bacterial abundance increased (Fig. 6B and D) and the microbiomes of both the small and large intestine became dominated by the administered species (*L. murinus* or *E. coli*) (Fig. 6A and C), with *L. johnsonii* remaining the second most abundant species. These results are consistent with our plate count results (Fig. 1G, 3D, 4G, and 7C and F) and support the conclusion that the observed probiotic effects primarily reflect the presence of the introduced strains rather than broad restructuring of the resident microbiota. They also align with a model in which probiotics act at least in part through niche exclusion (62), particularly in the case of EcN 1917, which directly competes with *K. pneumoniae* for resources such as oxygen (Fig. 2 and 3). Notably, however, the non-protective *L. murinus* strain ATCC 35020 (17) (Fig. 5A) also colonized both the large and small intestines efficiently (Fig. 6A and C), indicating that high-level colonization by an exogenous microbe alone is insufficient to confer probiotic protection.

### Dual species probiotic

Probiotic formulations are often administered as mixtures containing multiple strains, species, or even genera (63–65) under the rationale that their protective effects may be complementary: at a minimum additive, or in the best case synergistic. However, robust evidence for such interactions remains limited. We therefore sought to determine whether combining our two probiotic species of interest, EcN 1917 and *L. murinus* V10, would more significantly impact either the colonization of *Kp-39* in the neonatal intestine or survival against *Kp-43816*^lux^ sepsis relative to single-strain supplementation.

### The effect of dual-species (EcN 1917 and *L. murinus* V10) probiotic administration on *Kp-39* colonization is not additive and more closely mirrors *L. murinus* V10 supplementation

Littermate pups were administered either a dual-species probiotic consisting of EcN 1917 and *L. murinus* V10 or single-species probiotic controls prior to *Kp-39* challenge (Fig. 7). Dual-species supplementation significantly reduced total intestinal *Kp-39* colonization relative to both EcN 1917 (Fig. 7A) and *L. murinus* V10 (Fig. 7D) alone. Relative to EcN 1917 mono-supplementation, the dual-species probiotic produced greater reductions in *Kp-39* colonization burden in both the proximal and distal small intestine and in the colon (Fig. 7B). In contrast, when compared with *L. murinus* V10 alone, the only intestinal segment showing a statistically significant further reduction in *Kp-39* colonization burden under dual-species supplementation was the distal small intestine (Fig. 7E). Importantly, intestinal colonization levels of EcN and *L. murinus* V10 did not differ between single- and dual-species groups (Fig. 7C and F), indicating that neither probiotic strain impaired colonization by the other. Overall, these findings suggest that while dual-species administration can modestly enhance suppression of Kp-39 colonization, particularly relative to EcN 1917 alone, its effects more closely resemble those of *L. murinus* monotherapy than a strongly additive combination.

### The effect of dual-species (EcN 1917 and *L. murinus* V10) probiotic administration on survival of *K. pneumoniae* sepsis is not additive

To assess whether dual-species probiotic supplementation improved survival in the *Kp-43816*^lux^ sepsis model, litters of gentamicin-reared pups were simultaneously administered either *Kp-43816*^lux^ plus the dual-species probiotic or *Kp-43816*^lux^ with a PBS vehicle control and monitored daily for mortality (Fig. 7G). Dual-species probiotic supplementation significantly extended survival compared to the PBS vehicle control (Fig. 7H; *P* < 0.0001). However, survival outcomes for pups receiving the dual-species formulation did not differ significantly from those treated with either *L. murinus* V10 or EcN 1917 alone, indicating a lack of strong additive benefit. Indeed, the final survival rate among dual-species-treated pups (20%) was identical to that of those treated with *L. murinus* V10 alone and substantially lower than that of those receiving EcN 1917 alone (50% survival). These results suggest that, in this model, combining EcN 1917 with *L. murinus* V10 does not enhance protection against lethal *K. pneumoniae* sepsis beyond that afforded by the more protective single strain.

## DISCUSSION

Understanding the factors underlying microbial community assembly in the neonatal intestine is critically important as we seek to promote ecological conditions that favor the accumulation of symbiotic microbes while preventing the expansion of dysbiotic communities, both for disease prevention and for the mitigation of disease development.

### The neonatal mouse as a model for probiotic ecology and mechanism

Most studies of probiotic efficacy and mechanism have been conducted in adult animals or humans, where the intestinal microbiota are complex and mature, and where mechanistic inferences rely on indirect or relative measures of bacterial burden (for example, analysis of fecal contents or quantification of bacterial metabolites) (66, 67). In contrast, the work described here leverages the simplified community structure of the neonatal microbiome, baseline antibiotic “leveling” of the dams, and the delicate organismal size and tissue integrity of the neonatal mouse, which uniquely enable homogenization and quantitative assessment of the bacterial content of entire intestinal segments. Together, these features afforded a unique opportunity to directly interrogate the ecological principles underlying the activity of two probiotic species against the dysbiotic expansion of a clinically relevant pathogen. Our observation that priority effects can have major impacts on the effectiveness of specific probiotics exemplifies the power of this approach. Probiotics are commonly used prophylactically in clinical practice (68), yet it is often impossible to determine whether pathobionts are already present in premature human infants at the time of dosing. A limitation of our model is that we were unable to test the impact of probiotics on neonatal mice already infected with *K. pneumoniae*. We hypothesize that such a scenario would more closely resemble the simultaneous dosing regimen than the sequential one (Fig. 1 and 4), with outcomes likely depending on the extent to which the pathobiont has already established itself in the intestine. Moreover, not all cases of LOS are caused by *K. pneumoniae* (6), and our conclusions in this model may not extend to infections caused by other pathogens.

### EcN 1917 prevents both *Kp-39* dysbiosis and fulminant *Kp-43816*^lux^ sepsis

Our data indicate that EcN 1917 is the more effective probiotic in this neonatal model, reducing *K. pneumoniae* dysbiosis and substantially improving survival in fulminant sepsis. However, the use of a probiotic strain so closely related to pathogenic *E. coli* in a highly vulnerable patient population such as premature infants raises important safety considerations. Although extraintestinal infections caused by EcN 1917 appear to be rare in humans (69, 70), pathogenic *E. coli* strains are well established as major etiologic agents of a wide range of intra- and extra-intestinal infections (71–74). Notably, particularly in the context of this work, *E. coli* is among the primary species that cause neonatal sepsis, albeit this is an association most strongly tied to early- and not late-onset sepsis (75–77).

From an ecological perspective, probiotics administered in early life may act as pioneer species that can shape subsequent microbial succession (78). This is particularly true given the strong interspecies competition that occurs in the developing gastrointestinal tract (79). Persistent colonization by EcN 1917 could plausibly alter trajectories that otherwise define healthy microbiome development. Determining the duration of EcN 1917 colonization after neonatal supplementation, and its long-term effects on community composition and host outcomes, will be important for weighing immediate benefits against potential consequences for later-life ecology and disease risk.

Substantial safety and outcomes data will be needed before EcN 1917 is likely to be approved for use in human infants, particularly highly vulnerable pre-term infants, despite its apparent efficacy.

### Prevention of *Kp-43816*^lux^ sepsis

Although both *L. murinus* V10 and EcN 1917 effectively limited *Kp-39* dysbiosis, their protective capacities against *Kp-43816*^lux^ sepsis differed markedly. Because the *Kp-43816*^lux^ sepsis model required simultaneous administration of the probiotic and pathogen, at least part of this discrepancy may reflect *L. murinus* V10’s sensitivity to priority effects compared with EcN 1917 (Fig. 1 and 4). Additional work will be needed to determine whether, and to what extent, prior colonization with probiotics improves protection against sepsis in this and related models.

### More is not always merrier: variable effect of a dual-species EcN 1917-*L. murinus* V10 probiotic

Combining *L. murinus* V10 and EcN 1917 into a single dual-species probiotic enhanced protection against the development of *Kp-39* dysbiosis but did not translate to increased protection against the development of fulminant *Kp-43816*^lux^ sepsis (Fig. 7). While the rationale for multi-strain, multi-species, and even multi-genus probiotics is intuitively appealing, our data demonstrate that combining probiotic species does not necessarily result in additive or synergistic benefit. We can state this definitively only for the specific combination of *E. coli* Nissle 1917 and *L. murinus* V10 in the context of *K. pneumoniae* disease in our model. How other strains and species interact *in vivo* must be evaluated empirically, on a case-by-case basis. Even in the comparatively simplified neonatal intestine, there are complex microbial communities and the biology of multi-species interactions remains difficult to predict or infer *a priori*. Systematic testing of combinations of widely used probiotic strains across diverse models of probiotic action will be an important avenue for future research to determine how generalizable these phenomena are.

### Mechanisms of probiotic action in the neonatal intestine

The mechanistic underpinnings of EcN 1917 and *L. murinus* V10 probiotic action remain incompletely understood, and more broadly, the mechanism(s) by which probiotics contribute to neonatal health represent a major unanswered question in the field (10, 18–21, 26, 80). In this model, EcN 1917’s probiotic activity can be attributed in part to high-affinity oxygen respiration via CydA, consistent with the broader paradigm in which oxygen and other respiratory substrates are key determinants of pathobiont growth in the intestine (62, 81). Elucidating how these metabolic interactions translate into reduced sepsis-associated mortality (Fig. 3F) will be an important direction for future work. *L. murinus* V10 exhibits unusual oxygen-dependent phenotypes *in vitro* and exerts strain-specific probiotic effects against *Kp-39* dysbiosis in the proximal intestine *in vivo*, but these properties do not depend on its unique 110-kb megaplasmid, and the underlying molecular determinants remain unknown. In addition, the priority effect, specifically the dosing schedule of *L. murinus* V10 relative to *Kp-39*, appeared to modulate its protective capacity, whereas EcN 1917 efficacy was insensitive to the order of introduction of *L. murinus* V10.

We used transcriptomics to explore and identify potential mechanisms by which *L. murinus* V10 might exert its probiotic effects but have not yet pinpointed the molecular basis of its probiotic effect. A major limitation is the absence of robust genetic tools for *L. murinus*: as of April 2026, we are not aware of any published reports describing the generation of mutants of any *L. murinus* strain. We are actively working to develop genetic manipulation strategies for *L. murinus*, which will be essential for mechanistic dissection. From a translational standpoint, the rodent specificity of *L. murinus* (17, 44, 45) is another limitation. Once the mechanistic underpinnings of *L. murinus* V10 probiotic effects are better understood, our long-term goal is to apply those insights to the identification or engineering of related human probiotics, such as *Ligilactobacillus salivarius* (59), with the aim of developing improved therapeutic strategies for human neonates.

### Conclusions

In total, we have demonstrated how two disparate probiotic species colonize the murine neonatal intestine and interact with a clinically relevant pathogen in the context of neonatal dysbiosis. Collectively, our findings underscore the complex biological endeavor of probiotic intervention in the preterm intestine and the utility of applying community ecology as a framework for its analysis.

## MATERIALS AND METHODS

### Bacteria

#### Strains and plasmids used in this study

*E. coli* Nissle 1917 wild type was isolated from a commercial probiotic product (Mutaflor) (82). *E. coli* Nissle 1917 *cydA*, *cyoABCD, appC,* and *moaA* mutants were a gift from Sebastian Winter (University of California, Davis) (83). *K. pneumoniae* strain *Kp-39* (aka Xen 39) was obtained from PerkinElmer. *K. pneumoniae* strain *Kp-43816* (aka ATCC 43816) was obtained from the American Type Culture Collection and previously engineered to express bacterial luciferase (*Kp-43816*^lux^) (17). *E. coli* and *K. pneumoniae* strains were transformed with plasmids pWSK29 (*bla*^+^) or pWSK129 (*kan*^+^) (84) to confer ampicillin or kanamycin resistance, respectively. pWSK29 and pWSK129 are low-copy-number plasmids (6–8 copies per cell) derived from the pSC101 replicon and were specifically engineered for high segregational stability. Prior work has demonstrated that such low copy pSC101-based plasmids maintain excellent stability, particularly over short-to-moderate experimental time frames, with minimal spontaneous loss (84, 85).

*L. murinus* V10 was isolated from our mouse colony as previously described (17). *L. murinus* ATCC 35020 was obtained from the American Type Culture Collection. The identity of all strains was confirmed by whole-genome sequencing (SeqCenter, LLC), and the raw data are available on the NIH Sequence Read Archive (BioProject PRJNA979212).

#### Growth conditions

*K. pneumoniae* and *E. coli* strains were routinely grown aerobically on lysogeny broth (LB) medium (Difco) (86) at 37°C, with shaking for liquid cultures. *L. murinus* was grown on de Man-Rogosa-Sharpe (MRS) medium (Difco) (87). Aerobic liquid cultures of *L. murinus* were grown in 5 mL of MRS broth at 37°C with shaking. LB or MRS plates were prepared with 1.5% agar (Difco). Antibiotics were added as required: 100 µg mL^−1^ ampicillin, 50 µg mL^−1^ kanamycin, 12.5, 25, or 50 µg mL^−1^ rifampicin, or 5–30 µg mL^−1^ novobiocin. Anaerobic cultures were incubated at 37°C in a Coy anaerobic chamber containing 90% N_2_, 5% CO_2_, and 5% H_2_. Overnight cultures were typically grown for 16–20 hours.

#### Biosafety statement

All experiments involving bacteria were carried out using biosafety level 2 containment procedures, overseen by the UAB Institutional Biosafety Committee.

#### Generation of rifampicin-resistant *L. murinus*

*L. murinus* V10 was incubated anaerobically at 37°C for 48 h in MRS medium supplemented with 50 µg mL^−1^ rifampicin. Then, 100 µL of these cultures was spread on MRS plates supplemented with 25 µg mL^−1^ rifampicin and incubated anaerobically overnight at 37°C. Rifampicin-resistant colonies were isolated and genome sequenced by SeqCenter, LLC in Pittsburgh, PA, to ensure no off-target mutations occurred, and the resulting strain MJG2601 (a.k.a. *L. murinus* V10-Rif^R^), containing an H490Y substitution in RpoB, was used in experiments mapping *L. murinus* CFU *in vivo*. No difference in growth was observed between wild-type and Rif^R^ derivatives of *L. murinus* (data not shown).

#### Plasmid curing

Plasmid curing was performed using novobiocin as a curing agent at varying concentrations from 0 to 30 µg mL^−1^ (56, 57). Briefly, *L. murinus* V10 was incubated anaerobically overnight in 5 mL MRS broth, then subcultured into MRS broth containing various concentrations of novobiocin and incubated anaerobically for 72 h at 37°C. After 72 h of incubation, 100 µL was plated onto MRS agar and incubated overnight anaerobically at 37°C. Colonies were randomly selected and sent for genomic sequencing (SeqCenter, LLC) to confirm the absence of the pV10 plasmid, resulting in the pV10-free strain MJG2665, which had no other mutations relative to wild-type *L. murinus* V10.

### Mice

C57BL/6J mice were obtained from JAX room RB09, then immediately bred, and maintained at the University of Alabama at Birmingham (UAB). This colony was maintained only for the duration of the described experiments. All cages and cage bottoms were autoclaved prior to use, and mice were housed on standard chow (Lab Diet NIH-31 Tac Auto/Irradiated; 5L3Z) with sterile hydropacs as the sole water source. For experiments with antibiotic-treated females, timed pregnant littermates were co-housed for the duration of their pregnancies without males until embryonic day (E) 19. Fresh hydropacs were weighed, and gentamicin was added to a final concentration of 0.1 g L^−1^. Pregnant females were gavaged i.g. with a loading dose of 5 mg gentamicin. Antibiotics remained in the drinking water until p5. Two pregnant females who gave birth on the same day were co-housed with their pups through the remainder of the experiment. Pups were randomly assorted into treatment groups before their first treatment.

To maintain a high level of rigor, all of the mouse experiments described were pairwise littermate-matched experiments. Adding a third comparison group to any given experiment reduced the possible number of mice in each biological replicate to the point where loss of even a few pups (to maternal cannibalism, for example) could lead to failure of an entire experiment. In the figures showing the results of these experiments, each dot represents an individual mouse pup, and the sample size varies across comparisons because the number of available pups differed by litter and the number of independent replicates performed for each condition; no samples were pooled across experiments. Multiple litters were used for each experiment to minimize the possible impact of litter-to-litter variation in results.

### Competitive index determination

Five- or 14-day-old C57BL/6J wild-type mice were obtained from The Jackson Laboratory RB09. Mice were inoculated with 1 × 10^8^ CFU of each of the indicated bacterial strains (*E. coli* Nissle 1917 wild type and one mutant strain, each containing either pWSK29 or pWSK129 to confer differential antibiotic resistance), on p5 or p14. Mice were sacrificed 3 days after inoculation. Colons were removed, weighed, and homogenized in 1 mL PBS. Bacterial CFUs were then determined by plating serial 10-fold dilutions of the homogenates on selective media and incubating overnight at 37°C. Competitive indices were calculated as total CFU per colon of wild type divided by the total CFU per colon of mutant.

### *K. pneumoniae* dysbiosis and sepsis infection model

*K. pneumoniae* were grown aerobically overnight in LB (10 g L^−1^ Proteose Peptone No. 3, 5 g L^−1^ yeast extract, and 10 g L^−1^ NaCl) at 37 °C with shaking at 250 r.p.m. The following day, the culture was diluted 1:50 in fresh LB and incubated for 2–3 h to reach exponential growth phase. Bacteria were washed twice with sterile PBS and resuspended to deliver 10^7^ CFU per 50 μL dose i.g. via a 22-gauge flexible polypropylene gavage needle (Instech). For each experiment, a test dose was serially diluted and plated to confirm that between 5 × 10^6^ and 5 × 10^7^ CFU were delivered. Between each animal, the gavage needle was sanitized with 10% bleach and/or 70% ethanol, then dipped in sterile PBS for lubrication. Animals were imaged within 6 h of infection with the IVIS Lumina imaging system to visualize bioluminescent *K. pneumoniae*. Pups with a luminescence signal in the thoracic cavity were euthanized and removed from the experiment. Litters were observed one to two times daily throughout the remainder of each experiment. Septic pups were removed as soon as they were observed, and clean bedding was provided daily to prevent cannibalism and reduce the possibility of transmission of probiotic or pathobiont strains from pups to dams.

### Probiotic supplementation

#### Sequential dose probiotic model

Overnight anaerobic cultures of *Lactobacillus* strains grown in 5 mL of MRS broth were sub-cultured into 5 mL of fresh MRS broth, incubated anaerobically for 4 h at 37°C, and then washed and diluted with sterile PBS. EcN 1917 was grown aerobically overnight in 5 mL of LB broth and then sub-cultured in 5 mL of LB at 37°C with shaking at 200 rpm for 2.5 h before being washed and diluted with sterile PBS. A final dose of ~10^6^ CFU per 50 μL dose for each organism was gavaged i.g. on post-natal days p5 and p6. For each treatment, a test dose was serially diluted and plated on either MRS or LB agar, as appropriate, to confirm that between 1 × 10^5^ and 5 × 10^7^ CFU were delivered.

#### Simultaneous dose probiotic model

Growth conditions for each organism were the same as noted above. A final dose of 1 × 10^7^ CFU per 50 μL dose for each organism was gavaged i.g. on p7. For each treatment, a test dose was serially diluted and plated on either MRS or LB agar to confirm that between 5 × 10^6^ and 5 × 10^7^ CFU of each bacterium were delivered.

### Bacterial CFU determination

Complete intestinal segments were transferred to a pre-weighed microcentrifuge tube containing 1 mL of sterile PBS. Tissue was homogenized using a PowerGen500 homogenizer (Fisher), serially diluted in sterile PBS, and plated on LB agar containing 100 µg mL^−1^ ampicillin (EcN 1917) or LB agar containing 50 µg mL^−1^ kanamycin (*Kp-39*) and incubated overnight at 37°C in ambient air or on LB agar containing 100 µg mL^−1^ ampicillin (EcN 1917 Δ*cydA*) or MRS agar containing 12.5 µg mL^−1^ rifampicin (V10-Rif^R^) and incubated overnight at 37°C in a Coy anaerobic chamber in an atmosphere of 90% N_2_, 5% CO_2_, and 5% H_2_. The identity of *K. pneumoniae* colonies was confirmed by visual inspection in the dark to check for luciferase expression. Dilutions resulting in between 30 and 300 bacterial colonies per plate were recorded for colony counts the following morning. During homogenization, the homogenizer was cleaned with 70% ethanol and sterile PBS between samples. All CFU g^−1^ values are determined from plating the entire homogenate of each intestinal segment or appropriate dilutions thereof.

### Aggregation studies

*L. murinus* strains V10 and ATCC 35020 were incubated overnight at 37°C either aerobically or anaerobically in 5 mL MRS medium. The following morning, 100 µL of overnight culture was subcultured into 5 mL MRS medium and incubated for 5 h at 37°C either aerobically or anaerobically, then mixed by manual agitation and visually assessed for aggregation.

### mRNA sequencing

For RNA extraction, single colonies of *L. murinus* V10 were inoculated into 5 mL of MRS and grown either anaerobically overnight at 37°C or aerobically at 37°C with shaking at 200 r.p.m. Cultures were then harvested (three biological replicates for each condition), and RNA was extracted using the Qiagen RNeasy PowerMicrobiome Kit. mRNA sequencing was done by SeqCenter (Pittsburgh, PA). Samples were DNAse treated with Invitrogen DNase (RNase free). Library preparation was performed using Illumina’s Stranded Total RNA Prep Ligation with Ribo-Zero Plus kit and 10 bp unique dual indices (UDI). Sequencing was done on a NovaSeq X Plus, producing paired-end 150 bp reads. Demultiplexing, quality control, and adapter trimming were performed with bcl-convert (v4.1.5). Quality control and adapter trimming were performed with bcl-con-vert. Read mapping was performed with HISAT2. Read quantification was performed using Subread’s featureCounts functionality. Read counts loaded into R were normalized using edgeR’s Trimmed Mean of M values (TMM) algorithm. Subsequent values were then converted to counts per million (CPM). Differential expression analysis was performed using edgeR’s glmQLFTest.

### 16S rDNA sequencing

Large and small intestines were harvested on p7 (d0) from mice treated with PBS (*n* = 12), *L. murinus* V10 (*n* = 8), *E. coli* Nissle 1917 (*n* = 4), or *L. murinus* ATCC 35020 (*n* = 8) on days p5 and p6 (Fig. 1A and 4A). No *K. pneumoniae* was added to these mice. Tissue samples were stored at −80°C, then immersed in DNA/RNA Shield reagent (Zymo Research) and sent to Zymo Research for 16S amplicon sequencing. The ZymoBIOMICS-96 MagBead DNA Kit (Zymo Research, Irvine, CA) was used to extract DNA using an automated platform. Bacterial 16S ribosomal RNA gene targeted sequencing was performed using the Quick-16S NGS Library Prep Kit (Zymo Research, Irvine, CA). The bacterial 16S primers amplified the V3-V4 region of the 16S rRNA gene. The sequencing library was prepared using a library preparation process in which PCRs were performed in real-time PCR machines to control cycles and therefore limit PCR chimera formation. The final PCR products were quantified with qPCR fluorescence readings and pooled together based on equal molarity. The final pooled library was cleaned with the Select-a-Size DNA Clean & Concentrator (Zymo Research, Irvine, CA), then quantified with TapeStation (Agilent Technologies, Santa Clara, CA) and Qubit (Thermo Fisher Scientific, Waltham, WA). The ZymoBIOMICS Microbial Community Standard (Zymo Research, Irvine, CA) was used as a positive control for each DNA extraction. The ZymoBIOMICS Microbial Community DNA Standard (Zymo Research, Irvine, CA) was used as a positive control for each targeted library preparation. Negative controls (i.e., blank extraction control, blank library preparation control) were included to assess the level of bioburden carried by the wet-lab process. The final library was sequenced on Illumina Nextseq with a P1 reagent kit (600 cycles). The sequencing was performed with 30% PhiX spike-in. Unique amplicon sequence variants were inferred from raw reads using the DADA2 pipeline (88). Potential sequencing errors and chimeric sequences were also removed with the DADA2 pipeline. Taxonomy assignment was performed using Uclust from Qiime v.1.9.1 with the Zymo Research Database, a 16S database that is internally designed and curated, as reference. Composition visualization, alpha-diversity, and beta-diversity analyses were performed with QIIME v.1.9.1 (89). If applicable, taxonomy that has significant abundance among different groups was identified by LEfSe (90) using default settings. Other analyses, such as heatmaps, Taxa2ASV Deomposer, and PCoA plots, were performed with internal scripts. For absolute abundance quantification, a quantitative real-time PCR was set up with a standard curve. The standard curve was made with plasmid DNA containing one copy of the 16S gene and one copy of the fungal ITS2 region prepared in 10-fold serial dilutions. The primers used were the same as those used in targeted library preparation. The equation generated by the plasmid DNA standard curve was used to calculate the number of gene copies in the reaction for each sample. The PCR input volume was used to calculate the number of gene copies per microliter in each DNA sample.

### Statistical analysis

All statistical analyses were performed in GraphPad Prism 11. No assumption of normal distribution was made for any analysis, so nonparametric tests were used, including the Mann-Whitney test for pairwise comparisons, the Kruskal-Wallis test with Dunn’s multiple comparisons test for comparison of multiple treatments in experiments with a single independent variable, or a repeated measure two-way ANOVA with the Geisser-Greenhouse correction and Sídák’s multiple comparisons test for experiments with two independent variables. Kaplan-Meier survival curves were analyzed with the log-rank (Mantel-Cox) test.

## Data Availability

Raw data are available on the NIH Sequence Read Archive (BioProject PRJNA979212). All raw data for this paper not deposited in the NIH Sequence Read Archive as described above are available on the FigShare data repository (https://doi.org/10.6084/m9.figshare.c.8038144).

## SUPPLEMENTAL MATERIAL

Data Set S1 mbio.01127-26-s0001.xlsx RNA sequencing results of *Ligilactobacillus murinus* strain V10 grown with and without oxygen.

Data Set S2 mbio.01127-26-s0002.xlsx Relative abundance of reads from 16S rRNA amplicon sequencing in the small intestines of neonatal mice.

Data Set S3 mbio.01127-26-s0003.xlsx Relative abundance of reads from 16S rRNA amplicon sequencing in the large intestines of neonatal mice.

Supplemental Tables mbio.01127-26-s0004.pdf Tables S1 through S3.

ASM does not own the copyrights to Supplemental Material that may be linked to, or accessed through, an article. The authors have granted ASM a non-exclusive, world-wide license to publish the Supplemental Material files. Please contact the corresponding author directly for reuse.
