# Supplementary material for: Ecology of protection: probiotic biogeography and sepsis prevention in the neonatal intestine
Source: mBio. 2026 Aug 10;17(9):e01127-26. doi: 10.1128/mbio.01127-26 (PMC13556230; doi:10.1128/mbio.01127-26)
Supplement: Supplementary file 1 [file mbio.01127-26-s0004.pdf]

Ecology of protection: probiotic biogeography and sepsis prevention in the neonatal intestine

SUPPLEMENTAL INFORMATION

Sierra C. Hansen [a,b], Christopher W. Hamm [a], Jeffrey R. Singer [b,c], Casey T. Weaver [b, #], and Michael J. Gray [a, #]

[a] Department of Microbiology, Heersink School of Medicine, University of Alabama at Birmingham, Birmingham, AL 35294

[b] Department of Pathology, Heersink School of Medicine, University of Alabama at Birmingham, Birmingham, AL 35294

[c] Current address: Department of Pathology, Microbiology and Immunology, Vanderbilt University School of Medicine, Nashville, 37232

[#] Address correspondence to Michael J. Gray, mjgray@uab.edu, or Casey T. Weaver, cweaver@uabmc.edu

SUPPLEMENTAL TABLES

Locus Tag	Predicted Function of Gene Product	Log_2-Fold Change
FEE40_13325	hypothetical protein	8.45
FEE40_13030	DUF739 family protein	6.55
FEE40_04875	PTS sugar transporter subunit IIC	6.40
FEE40_04885	PTS fructose transporter subunit IIA	6.05
FEE40_04880	PTS system mannose/fructose/sorbose family transporter subunit IID	5.99
FEE40_12565	lipoate--protein ligase	5.96
FEE40_04890	SIS domain-containing protein	5.95
FEE40_12575	2-oxo acid dehydrogenase subunit E2	5.94
FEE40_12570	dihydrolipoyl dehydrogenase	5.66
FEE40_12900	hypothetical protein	5.60
FEE40_12580	alpha-ketoacid dehydrogenase subunit beta	5.51
FEE40_12585	pyruvate dehydrogenase (acetyl-transferring) E1 component subunit alpha	4.71
FEE40_12860	hypothetical protein	4.58
FEE40_11085	DUF871 domain-containing protein	4.56
FEE40_05810	LysM peptidoglycan-binding domain containing protein	4.55
FEE40_03440	YSIRK-type signal peptide-containing protein	4.43
FEE40_04870	PTS sugar transporter subunit IIB	4.34
FEE40_04715	thioredoxin-disulfide reductase	4.21
FEE40_07885	ABC transporter permease	4.19

FEE40_01715	FAD-dependent oxidoreductase	4.12

Supplemental Table S1. The twenty most up-regulated genes in aerobically grown *L. murinus* strain V10. Predicted functions are listed for each gene, when available. 400 genes were significantly more highly expressed in aerobically-grown cultures of *L. murinus* strain V10 than in anaerobically-grown cultures, with log_2-fold changes in gene expression > 1. See Supplemental Data Set 1 for complete RNA sequencing results.

Locus Tag	Predicted Function of Gene Product	Log_2-Fold Change
FEE40_02435	ATP-binding cassette domain-containing protein	-8.15
FEE40_00400	APC family permease	-5.71
FEE40_02515	ACP phosphodiesterase	-4.80
FEE40_06155	IS256 family transposase	-4.54
FEE40_06425	amino acid permease	-4.30
FEE40_04720	hypothetical protein	-4.26
FEE40_06335	aquaporin family protein	-4.09
FEE40_07000	MFS transporter	-3.95
FEE40_03925	sugar transferase	-3.83
FEE40_01235	hypothetical protein	-3.63
FEE40_06925	glutamate/gamma-aminobutyrate family transporter YjeM	-3.57
FEE40_06310	glucose transporter GlcU	-3.57
FEE40_12265	aldo/keto reductase	-3.55

FEE40_12240	IS200/IS605 family element transposase accessory protein TnpB	-3.49
FEE40_04380	NCS2 family permease	-3.41
FEE40_05140	ECF transporter S component	-3.31
FEE40_12830	acytransferase	-3.29
FEE40_04055	ATP-binding cassette domain-containing protein	-3.28
FEE40_05145	nucleoside hydrolase	-3.27
FEE40_04080	ABC transporter ATP-binding protein	-3.20

Supplemental Table S2. The twenty most down-regulated genes in aerobically grown *L. murinus* strain V10. Predicted functions are listed for each gene, when available. 415 genes were significantly less highly expressed in aerobically-grown cultures of *L. murinus* strain V10 than in anaerobically-grown cultures, with log_2-fold changes in gene expression < -1. See Supplemental Data Set 1 for complete RNA sequencing results.

**Locus Tags**		**Predicted Function of**	
**V10**	**ATCC 35020**	**Gene Product**	**LogFC**
FEE40_05810	EDJADAIO_00467	LysM peptidoglycan-binding domain-containing protein (232aa possible aggregation promoting factor)	4.55
FEE40_03440	EDJADAIO_01139	YSIRK-type signal peptide-containing protein (1702aa	4.43

		Rib/alpha ClfA clumping factor)	
FEE40_06275	EDJADAIO_00381	LPXTG cell wall anchor domain-containing protein (5,673aa MucBP mucin binding protein)	4.02
FEE40_00120	none	YSIRK-type signal peptide-containing protein (750aa Rib/alpha repeat protein)	3.73
FEE40_05410	EDJADAIO_00497	LysM peptidoglycan-binding domain-containing protein (199aa lysozyme-like domain protein)	3.54
FEE40_05300	EDJADAIO_00521	YSIRK-type signal peptide-containing protein (2946aa MucBP / PspC / FhaB domain protein)	3.41
FEE40_07850	none	YSIRK-type signal peptide-containing protein (1620aa MucBP-domain adhesion protein)	2.89
FEE40_04050	EDJADAIO_00763	LPXTG cell wall anchor domain-containing protein	2.81

		(3278aa MucBP mucin binding / aggregation substance domain protein)	
FEE40_03180	none	LPXTG cell wall anchor domain-containing protein (71aa hypothetical protein)	2.26
FEE40_02175	EDJADAIO_00319	LysM peptidoglycan-binding domain-containing protein (432aa predicted endopeptidase)	2.23
FEE40_12415	EDJADAIO_01139	YSIRK-type signal peptide-containing protein (3103aa Rib/alpha ClfA clumping factor)	2.14
FEE40_00105	EDJADAIO_01417	LPXTG cell wall anchor domain-containing protein (2500aa Rib/alpha repeat protein)	2.08
FEE40_08485	none	LysM peptidoglycan-binding domain-containing protein (166aa hypothetical protein)	2.08
FEE40_08580	none	LysM peptidoglycan-binding domain-containing protein	1.82

		(429aa autolysin / muramidase)	
FEE40_03445	EDJADAIO_01139	YSIRK-type signal peptide-containing protein (2031aa Rib/alpha ClfA clumping factor)	1.53

Supplemental Table S3. Comparison of expression of selected surface proteins from *L. murinus* strains V10 and ATCC 35020. Fifteen predicted surface proteins from *L. murinus* V10 that showed > 1.5 $\log_2$-fold increases in expression in aerobically grown cells, along with the locus tags of the most closely homologous gene in *L. murinus* strain ATCC 35020, if a close homolog was present in that strain. These genes are the same ones indicated with black dots in Figure 5B. Note that for three of the predicted surface proteins of *L. murinus* V10 (encoded by FEE40_03440, FEE40_12415, and FEE40_03445), the closest homolog in *L. murinus* ATCC 35020 was the protein encoded by EDJADAIO_01139. See Supplemental Data Set 1 for complete RNA sequencing results.
